# Eleven-Year Trend of Drug and Chemical Substance Overdose at a Local Emergency Hospital in Japan

**DOI:** 10.7759/cureus.32475

**Published:** 2022-12-13

**Authors:** Takanao Hashimoto, Yudai Kaneda, Akihiko Ozaki, Arinobu Hori, Takashi Tsuchiya

**Affiliations:** 1 Pharmacy, Kenkodo Pharmacy, Osaki, JPN; 2 School of Medicine, Hokkaido University, Sapporo, JPN; 3 Breast Surgery, Jyoban Hospital, Tokiwa Foundation, Iwaki, JPN; 4 Psychiatry, Hori Mental Clinic, Minamisoma, JPN; 5 Surgery, Sendai City Medical Center, Sendai, JPN

**Keywords:** social support, psychiatry, emergency, over-the-counter drug, overdose

## Abstract

Purpose: The purpose of this study was to investigate long-term trends of overdose in the emergency department of a regional core hospital in Sendai, Miyagi Prefecture, Japan, and to identify patient characteristics as well as drugs and chemicals associated with overdose.

Methods: Patients who visited the emergency department from January 1, 2010, to December 31, 2020, and were diagnosed with a drug or chemical overdose were included in the study. We conducted a descriptive analysis based on the data collected.

Results: In total, 577 patients (mean 38.4 years old, female 75.0%) were considered, and 16.8% had a history of repeated overdose. The number of patients during the study period showed a downward trend, with slight increases in 2012 and 2020. In addition, the top four drugs suspected of causing overdose were over the counter (OTC) antipyretic analgesics and cold medicines (N=97), followed by flunitrazepam (N=80), etizolam (N=72), and brotizolam (N=70).

Conclusion: There was a decreasing trend in overdose, and OTC medicines, sedatives, and anxiolytics were the primary medications causing overdose. OTC antipyretic analgesics and cold medicines were the most common suspected overdose drugs, with an increasing trend in the later years.

## Introduction

Drug overdose is a serious health problem worldwide. In Japan, the National Center of Neurology and Psychiatry surveyed 2,733 inpatients at more than 1,200 hospitals to determine the situation of patients with drug-related psychiatric diseases [1]. The most commonly reported drugs that patients had used at least once in their lifetime were stimulants (N=1748), sedatives/anxiolytics (N=935), volatile solvents (N=911), marijuana (N=845), over-the-counter (OTC) drugs (N=429), and legal highs (N=409) [1]. However, there are very few medical institutions in Japan that treat patients with drug-induced psychiatric diseases, and it is still questionable whether these results reflect the reality of overdoses.

In Japan, psychiatrists play a crucial role in the treatment of overdoses; in EDs that accept overdose patients, physical symptoms are treated by all healthcare providers, but psychiatric symptoms are taken into account and followed up by psychiatrists from a long-term perspective [2]. Psychiatrists also identify suspected overdose medications and causes of overdoses such as abuse or suicide [2]. There have been studies that have examined the relationship between the dose of suspected overdose medications and the need for psychiatric hospitalization [3], and the relationship between dose and severity of overdose [4], all of which involved psychiatrists as members of the ED. On the other hand, some local medical facilities with ED have to accept overdose patients even in the absence of a psychiatrist. In this context, a previous study published in 2008, which recruited 194 overdose patients for three years, reported that the most suspected drugs were benzodiazepines [5]. No other studies have investigated the actual situation of overdose patients in an ED without a psychiatrist.

Sendai City Medical Center, located in Sendai City, Miyagi Prefecture, Japan, is the only secondary emergency medical institution in the system of the Sendai Emergency Medical Service Foundation [6]. This center accepts referrals from the Sendai Emergency Medical Center, a primary emergency medical center, and local clinics, as well as direct emergency visits from patients including emergency transport. Therefore, the Sendai City Medical Center has also played an important role as a receiving center for overdose patients, even without a specialized department for psychiatry or drug addiction. In this study, we retrospectively investigated overdose patients who visited the ED of Sendai City Medical Center from 2010 to 2020.

## Materials and methods

Details of overdose patients who had visited the ED of Sendai City Medical Center, Sendai, Japan, between January 1, 2010, and December 31, 2020, were extracted from the electronic medical records by searching the patients whose diagnosis included terms such as "drug overdose", “psychotropic overdose”, or “chemical substance overdose”. A wide range of cases of exposure to chemical components, not just typical drug overdose was collected. As a result, 577 overdose patients were examined in this study.

The following data were extracted from medical records: age, sex, date and time of visit, Japan Coma Scale, overdose drugs, reasons for overdose, history of repeated overdose, suicide attempts, drinking status, history of psychiatric diseases, and outcome (discharge, hospitalization, transfer to a higher medical institution, or death). The diagnosis of overdose drugs was made by interviewing the patient and his associates, by checking the actual medications they had brought, or by qualitative examination using urine. For the dose of the suspected drugs, we simply calculated the number of units and did not calculate the dose if the amount of medication was unknown or could not be calculated (liquid or powder). The reasons for overdose were categorized based on the suicide statistics of the National Police Agency [7], a previous study [8], and motives related to family, health, economy, employment, romance, and school. OTC antipyretic analgesics and cold medicines were defined as OTC medicines containing one or more of the following ingredients: ibuprofen, acetaminophen, acetylsalicylic acid, dihydrocodeine, pseudoephedrine, and/or methyl ephedrine. Detergents were defined as household cleaners for kitchen, bath, or toilet use.

We expressed the study characteristics of overdose patients as mean ± standard deviation or percentages. The number of overdose patients was categorized by year, month, and day of the week. We did descriptive analyses for the obtained data. This study was approved by the Institutional Review Board of Sendai City Medical Center (approval number: 2020-0049).

## Results

The basic characteristics of the 577 overdose patients are shown in Table 1. The mean age of the patients was 38 years and 75.0% were women. The average number of concomitant drugs was 2.8±2.2, with 63.6% of patients overdosing on two or more drugs. The most common reasons for overdose were related to health (51.8%), followed by family (6.6%), and employment (2.6%).

**Table 1:** Demographic characteristics of overdose patients (N=577) Variables showed mean ∓ standard deviation or percentage (number of patients) *Patients missing data were excluded.

Age, years	
Mean	38.4 ∓ 17.5
Median (minimum-maximum)	34.0 (14-95)
Age group, % (N)	
0-9 years	0 (0)
10-19 years	7.6 (44)
20-29 years	30.7 (177)
30-39 years	25.1 (145)
40-49 years	14.4 (83)
50-59 years	9.0 (52)
60 years and above	13.2 (76)
Sex, Female, % (N)	75.0 (433)
Japan Coma Scale (N=550*), % (N)	
0	30.2 (166)
1-3	34.9 (192)
10-30	19.3 (106)
100-300	15.6 (86)
Combination of drug (N=546*)	
Mean	2.8 ∓ 2.2
Median (minimum-maximum)	2.0 (1-21)
2 types≤, % (N)	63.6 (347)
Dose, tablet/pack (N=464*)	
Mean	58.5 ∓ 60.1
Median (minimum-maximum)	40.0 (1-640)
Reasons, % (N)	
Family-related	6.6 (38)
Health-related	51.8 (299)
Economy-related	0 (0)
Employment-related	2.6 (15)
Romance-related	1.9 (11)
School-related	1.0 (6)
Unknown	36.0 (208)
History of repeat overdose, % (N)	16.8 (97)
Suicide attempt, % (N)	19.4 (112)
Drinking alcohol, % (N)	14.6 (84)
History of psychiatric diseases, % (N)	63.3 (365)
Outcome, % (N)	
Discharge	47.7 (275)
Hospitalization	68.6 (396)
Higher hospital transfer	0.9 (5)
Death	0.2 (1)

**Figure 1:**
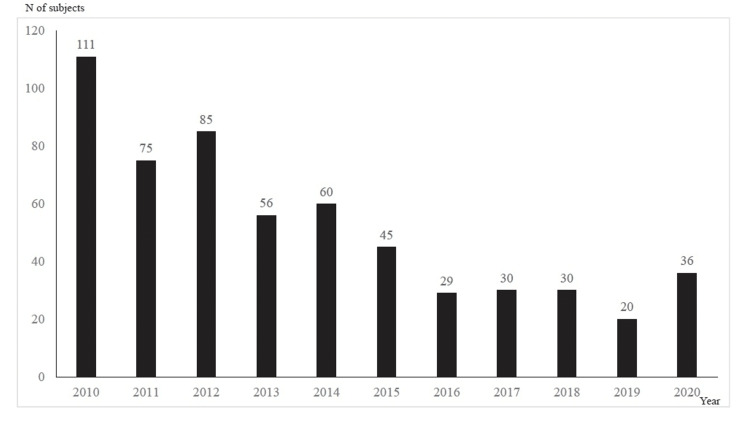
Annual change in the number of overdose patients It continued to decline from 2010 to 2019 overall, but turned to increase in 2020.

**Figure 2:**
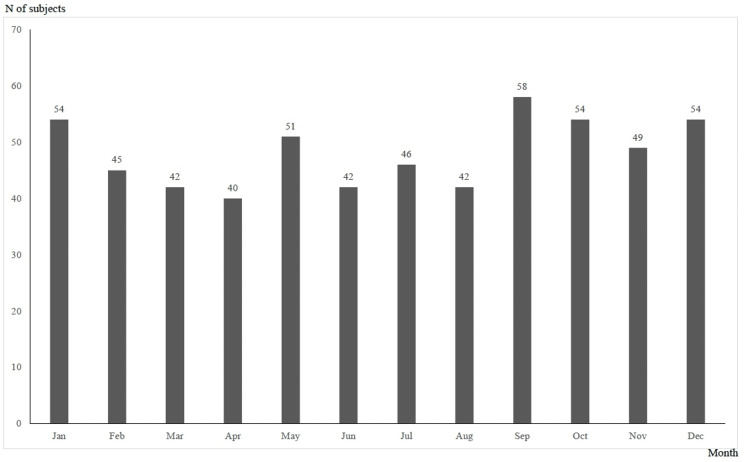
Overdose patients by month The month of visit to the emergency department of Sendai City Medical Center was counted

**Figure 3:**
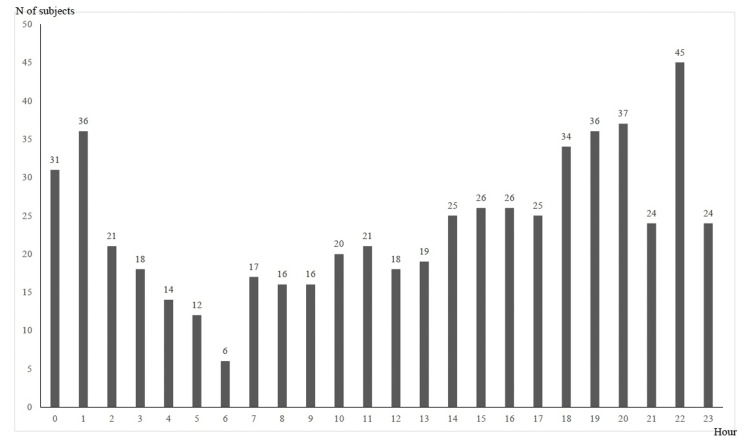
Overdose patients by 24 hours The time of visit to the emergency department of Sendai City Medical Center was counted

**Figure 4:**
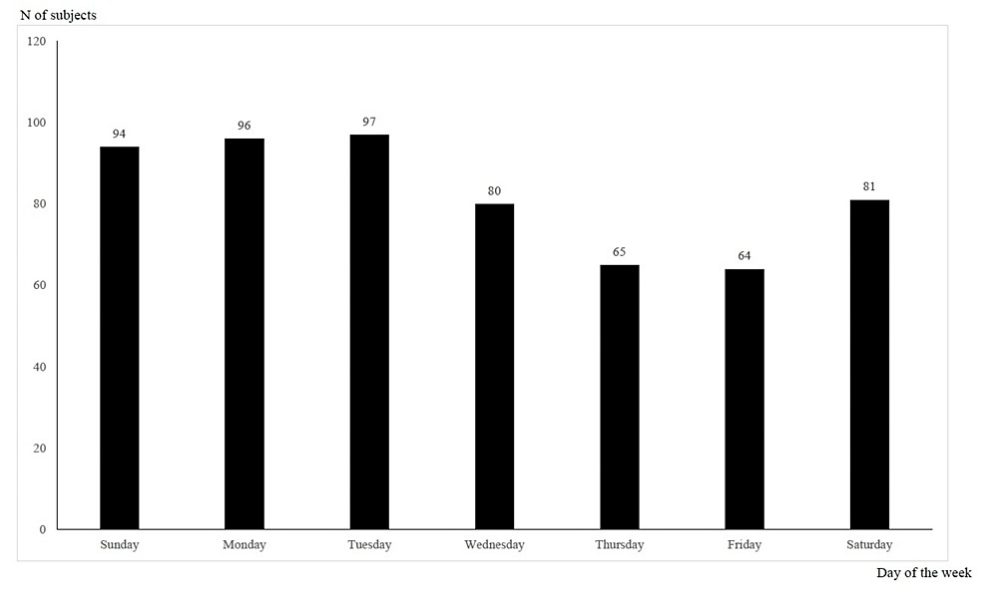
Overdose patients by day of the week The day of the week of visit to the emergency department at Sendai City Medical Center was counted

Figure 1 shows the temporary trend in the number of overdose patients from 2010 to 2020; the number of patients peaked in 2010 and was on a decreasing tendency until 2019, but it turned to increase in 2020. Categorized by month, 58 patients were examined in September, 54 in January, October, and December, and 51 in May (Figure 2). Categorized by 24-hour period, 45 patients were at 22:00, 37 at 20:00, and 36 at 1:00 and 19:00 (Figure 3). Categorized by day of the week, 97 patients were examined on Tuesday, 96 on Monday, and 94 on Sunday (Figure 4).

**Figure 5:**
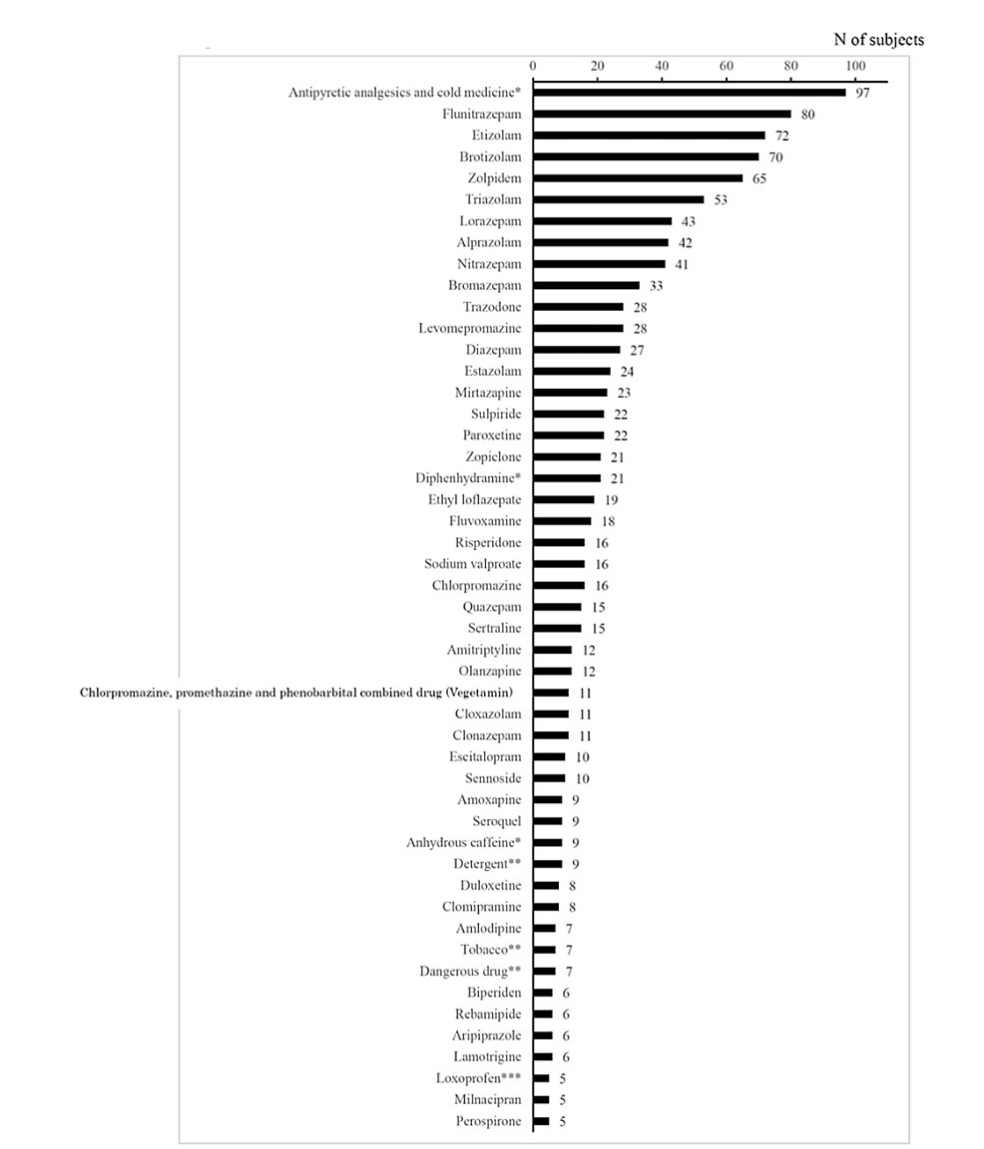
List of drugs that were overdosed on by more than five patients *Over-the-counter (OTC) drugs; ** Daily products and legal highs; *** Mixture of prescription and OTC drugs

Figure 5 shows overdose drugs that five or more patients had taken. The highest number was 97 for OTC antipyretic analgesics and cold medicines, followed by 80 for flunitrazepam and 72 for etizolam. Among the other OTC drugs, it was 21 for diphenhydramine and nine for anhydrous caffeine. In addition, there were nine patients for detergents and seven for tobacco and legal highs. Figure 5 also showed that 11 patients took chlorpromazine, promethazine, and phenobarbital combined drugs (Vegetamin). In addition, two and one patients overdosed on amobarbital and pentobarbital, respectively. Notable overdose drugs that are not listed in Figure 5 were OTC bromvalerylurea (N=4), opioid (morphine) (N=1), and marijuana (N=1). There were three patients for organophosphorus substances including insecticides or pesticides. Also, one patient was found to have automobile exhaust gas poisoning due to a suicide attempt.

Table 2 shows the 11-year overdose trends in OTC antipyretic analgesics and cold medicines and the top three prescription drugs overdosed by more than five people (as shown in Figure 5). While flunitrazepam was the most common drug in 2010 and 2011, OTC antipyretic analgesics and cold medicines were the most common in 2012-15, 2019, and 2020.

**Table 2:** Eleven-year trends in OTC antipyretic analgesics and cold medicine, and the top three prescription drugs overdosed on by more than five people * indicates the most common drugs in each year. OTC: over the counter

	2010	2011	2012	2013	2014	2015	2016	2017	2018	2019	2020
All	111	75	85	56	60	45	29	30	30	20	36
OTC antipyretic analgesics and cold medicine, % (N)	13.5 (15)	14.7 (11)	18.8 (16)*	16.1 (9)*	23.3 (14)*	24.4 (11)*	6.9 (2)	10.0 (3)	6.7 (2)	40.0 (8)*	16.7 (6)*
Flunitrazepam % (N)	16.2 (18)*	20.0 (15)*	14.1 (12)	12.5 (7)	11.7 (7)	8.9 (4)	17.2 (5)*	10.0 (3)	6.7 (2)	25.0 (5)	5.6 (2)
Etizolam % (N)	13.5 (15)	20.0 (15)*	8.2 (7)	8.9 (5)	10.0 (6)	17.8 (8)	13.8 (4)	13.3 (4)	6.7 (2)	5.0 (1)	13.9 (5)
Brotizolam % (N)	15.3 (17)	9.3 (7)	14.1 (12)	12.5 (7)	6.7 (4)	6.7 (3)	17.2 (5)*	20.0 (6)*	16.7 (5)*	5.0 (1)	8.3 (3)

The number of overdose OTC drug brands included 24 brands for antipyretic analgesics and cold medicines, six brands for diphenhydramine (five for sleep improvement and one for motion sickness), and one for anhydrous caffeine. The number of overdose patients of a combination of two or more OTC drugs with the same effect were observed in 19 patients for antipyretic analgesics and cold medicines (15 patients for two types, three patients for three types, and one patient for four types) and in five patients for diphenhydramine (four patients for two types and one patient for three types).

## Discussion

This study revealed the long-term trend of overdosing over 11 years and the characteristics of overdose patients in a secondary emergency medical hospital without a psychiatry department in Sendai, Miyagi, Japan. Overall, the number of overdose patients was a decreasing trend in the study period.

First, the reason for the downward trend in the number of overdose patients during the study period may be related to various efforts to promote the safe use of medicines in Japan. In 2010, the Ministry of Health, Labour and Welfare (MHLW) organized the Suicide and Depression Project Team to launch overdose measures to reduce the number of suicides, which at that time exceeded 30,000 per year [9]. One of the causes of overdoses is the prescription of multiple psychiatric drugs in medical institutions, and measures have been taken to ensure appropriate drug prescribing. Second, there are regulations on the proper use of psychotropic drugs. Vegetamine (a combination of chlorpromazine, promethazine, and phenobarbital) was removed in 2016 to promote the safer use of psychiatric medications. Additionally, etizolam and zopiclone were listed as psychotropic drugs in 2016, limiting the number of days of prescription to 30 days or less. Third, the system against illegal drugs has improved. In Japan, the use of drugs containing hallucinogenic ingredients, known as "legal herbs," increased from around 2009 to 2012 [10]. Under Japanese law, drugs are regulated by their chemical structure, and even a partial change in chemical structure can result in a drug being exempt from regulation. Therefore, in 2012, 17 new chemical substances were added to the list of controlled chemicals [10], and in 2014, MHLW and the National Police Agency collaborated to rename "legal herbs" to "law-evading herbs" and then to "legal highs" (called “dangerous drug” in Japan) and continued the countermeasures [11]. Although the number of overdose patients for legal highs in this study is relatively small (N=7), it is suggested that the growing interest in overdoses in Japan is related to the decrease in the number of patients. Therefore, various overdose countermeasures implemented by the government and medical institutions after 2010 may have contributed to the decrease in the number of overdose patients. Pesticide poisoning might be a phenomenon unique to Japan among developed countries. However, since the 1980s, several pesticides, including paraquat, have been regulated by law, and since then the number of deaths has been declining [12].

This study found that overdoses for relatively commonly available medicines, such as OTC drugs, have increased, especially since 2012 (Table 2). In addition, 24 patients overdosed on multiple brands of OTC drugs. In both studies conducted in 2004 [5], and 2010-12 [4], the most common drugs were benzodiazepines, followed by antipsychotics. However, the tendencies of overdose may be changing recently. In fact, recent reports have shown an increase in the percentage of OTC drugs in suspected overdoses [1]. These changes in the overdose situation can be explained by the fact that it has become easier to purchase OTC drugs for the following reasons: (i) loxoprofen sodium was switched to OTC in 2011, (ii) internet sales of many OTC drugs such as antipyretic analgesics and cold medicines were approved in 2014, and (iii) the self-medication tax system was introduced in 2017 to refund or reduce a certain amount of tax by purchasing OTC drugs. However, a study by MHLW has revealed that the environment for the proper and safe purchase of OTC drugs may still be inadequate [13]. According to a survey of 5,000 pharmacies on the sale of OTC drugs, it was seen that 53% of them let customers purchase several potentially abusive OTC drugs without asking any questions [13]. As for the Internet sales of OTC drugs, one site in Japan allowed customers to make purchases by simply answering a self-administered questionnaire. What the MHLW had initially envisioned was a system of internet sales of OTC drugs based on two-way communication between pharmacists and customers through the provision of safety information via e-mail or telephone [14].

In our study, there was only one death due to overdose. In Japan, studies on the association between overdose and death are very limited. In a study of 538 patients with overdose who were transported to tertiary emergency hospitals, there were three deaths due to overdose [4]. According to a study that examined the reasons for 81 men and women who attempted suicide in 2009, overdose was the most common with 57% (N=46), among which benzodiazepines were the most common [15]. A study of 359 autopsies conducted between 2011 and 2015 found 12 cases of synthetic cathinone/cannabinoid users and 10 methamphetamine users [16]. In addition, overdose deaths have been reported as case reports for methamphetamine [17], diphenhydramine [18], and Brotizolam [19]. On the other hand, a study of opioid overdose deaths in the United States from 2001 to 2017 found that there were more than 380,000 deaths over the entire period, and the number of deaths in 2017 was about 4.6 times higher than in 2001 [20]. In Canada, the number of deaths per 100,000 from drug overdose has increased from 6.6 in 2011 to 43.6 in 2021 [21]. The drug with the highest number of deaths was fentanyl, followed by cocaine [21]. In Norway, the report showed that the push for an opioid prescription policy has increased pharmaceutical opioid dispensing, but also increased overdose deaths [22]. These findings suggested that deaths due to drug overdose could be mainly associated with opioids. According to data from a survey of drug use in 2010, morphine consumption per million people per day was 430 g in Austria, 205 g in the United States, and 192 g in Canada, compared to 7 g in Japan [23]. Thus, it was suggested that the low use of opioids in Japan might be related to the low number of drug overdose deaths.

As mentioned above, the mortality rate of overdose patients in Japan is low. These facts may suggest that providing long-term social support to overdose patients is very important. At Sendai City Medical Center, we only focus on treating the physical symptoms of overdose patients. However, the current support system for overdose patients in Japanese local governments may be inadequate. For example, the holiday and night counselling service for psychiatric emergencies does not cover alcohol use disorder and overdose patients [24]. The overdose prevention policy in Japan focuses more on keeping people from getting involved in crimes rather than directly helping overdosed patients [25]. There is no psychiatric support system in EDs at hospitals without psychiatric departments.

According to the tendencies of overdose incidents by month, time of day, and day of the week (Figures 2, 3, 4), there is an increase at night, in the first half of the week, and in January, May, and September to December. The National Police Agency's monthly figures on suicides show that the number of suicides is high in March, May, and October [7]. A study of suicide tendencies over the past 41 years has shown that there are more suicides on Mondays and after holidays in the early morning and afternoon than at night [26]. In contrast, another survey found that the most common time for overdose is at night [5]. The results of our study were similar to previous studies in terms of monthly and daily trends in overdose, but the results were not consistent in terms of the time of day.

There are several limitations in this study. First, this study was conducted at a single institution, so we do not know if the results can be applied to the entire population of Japan. Second, no differential diagnosis based on laboratory or quantitative examination has been made. Although qualitative examination with test kits has been performed for some drugs, treatment of overdose had been based on a physician's diagnosis based on interviews with patients and their relations and/or on confirmation of the actual drug. The dose of an overdose drug is important in investigating the symptoms and their severities. We examined the dose data listed in Table 1. However, since the main focus of our study was to determine the distribution of drug types, we did not examine the doses in any further detail. Further, medical records filled at the ED often did not include the extent of drugs taken by patients, which made it difficult for us to make a thorough data collection about this matter. As a result, underestimation may exist. Third, the number of patients with a history of mental disorders was 63.3%, which may have been overestimated. This is probably due to the fact that diagnosing a mental illness is covered by insurance.

## Conclusions

As an overall trend, there was a decrease in overdoses, possibly due to Japan's overdose countermeasures since 2010. OTC antipyretic analgesics and cold medicines were the most common suspected overdose drugs, with an increasing trend in the latter half of the study period. Although countermeasures against psychiatric drugs have been strengthened since 2010, the regulation of the sale of OTC drugs seems to be inadequate. It is important to establish a system for the proper use of OTC drugs.
